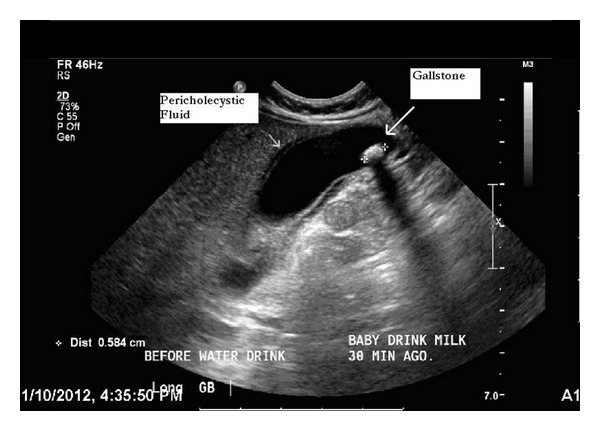# An Unusual Cause of Vomiting in an Infant of 3 Months of Age

**DOI:** 10.1155/2012/913481

**Published:** 2012-04-08

**Authors:** Kumara Nibhanipudi, Akram Al-Husaini, Samrina Kahlon, Richard K. Stone

**Affiliations:** NYMC, Metropolitan Hospital Center, USA

## Abstract

This is a 3-month-old infant with a diagnosis of acute cholecystitis presenting with projectile vomiting and no fever and no abdominal pain.

## 1. Case Report

A 3-month-old infant has come for complaint of vomiting with one day: no fever, no diarrhea, no history of trauma.

V.S. temp 98.F rectal. HR 142R.R. 34; B/P 86/29. Maternal history: no history of diabetes, or gall stones in the family, 25 yrs old, G1, P0 with uneventful pregnancy. Birth history: FT. C/S secondary to nonreassuring fetal heart rate LGA. Birth weight of 4.1 kg. Nursery: uneventful; new born screen is negative. Blood group baby O +ve; mother O +ve and coombs negative. And patient was discharged with the mother on 2nd day of life. Feeding history: breast feeding with supplemental bottle feeding similac with Fe q 3 hr. Bowel movements: one bowel movement this morning. Soft pasty stools. Family history: unremarkable. PMHx: at 13 days of age: treated for omphalitis, with amoxicillin.

On PE: this is a 3-month-old infant well developed well nourished. Not in distress. Ant. Front. Flat. Eyes; PERL; red reflex is present. Pt not jaundiced. Ears: TMs intact. No aural discharge. Chest: clear. Heart sounds are normal no murmurs. Abd: not distended. No olive is felt and no visible peristalsis. No tenderness is noted and no organomegaly. Hernias sites are within normal limits. Stools guaiac is negative. Imp: in view of the projectile vomiting, patient was sent to ultrasound to rule out pyloric stenosis. Plan: patient was sent to ultrasound, found to have a mobile gall stone 5 mm with dilated common bile duct measuring 3 mm in diameter and pericholecystic fluid suggestive of cholecystitis (Figure 1).

Plan admitted to pediatric floor for further workup. The patient was observed in the pediatric floor and sent home to be flowed without surgical intervention at present with the notion that this stone may pass off uneventfully.

## 2. Discussion

We are describing an unusual case of calculous cholecystitis in infant 3 months of age, with an apparent normal life and also without prior history of cholestasis. According to Debray et al. [1], 40 infants were found to have cholelithiasis during 17-year period. In 6 infants they were found to have incidental findings on sonogram with no signs of common bile duct obstruction. Spontaneous resolution of cholelithasis occurred in 25 patients.

The formation of gall stones is by supersaturating of bile either by cholesterol or by bilirubin, followed by formation of nucleation of cholesterol and finally status of gall bladder allowing formation of calcium bilirubinate crystals which lead to gall stone. Infection by process of deconjugation of bilirubin glucronide increases the concentration of unconjugated bilirubin in the bile which predisposes to the formation of the gall stone. Other common causes are hemolytic diseases, malabsorption, necrotizing enterocolitis and hepatobiliary disease, multiple blood transfusions, Cystic fibrosis, phototherapy, polycythemia, and hyperalimentation. In USA, the most common infections associated with cholecystitis are staphylococcus, Enterobacter, Citrobacter, Salmonella species, chronic urinary tract infections, and Ascariasis.

 The patient usually complain of colicky abdominal pain or pain localized to the right upper quadrant and sometimes radiating to shoulder or back. Anorexia, nausea, and vomiting are common complaints in acute attack. Sometimes followed by jaundice or dark urine. Fever and chills are uncommon. In our patient, the infant had no fever, and only vomiting with no complaint of colicky abdominal pain and also patient did not have any tenderness over the abdomen or over the right upper quadrant. Our patient had slightly elevated liver enzymes with normal bilirubin and urine analysis did not show any bilirubin.

Our patient has no evidence to suggest any hemolysis, nor was he given any TPN, blood transfusions, any phototherapy.

 As per Jacir and others [2], spontaneous resolution of gall stones has been observed in 3 out of 4 infants. And the surgery team in our hospital decided to follow up without surgery at present.

## 3. Conclusions

This is an unusual case of calculous cholecystitis presenting only as vomiting and no abdominal pain or fever.

## Figures and Tables

**Figure 1:**